# Farm to Early Care and Education Programming: A Descriptive Study of Challenges and Opportunities to Promote Healthful Foods to Young Children

**DOI:** 10.3390/ijerph17186857

**Published:** 2020-09-19

**Authors:** Morgan L. McCloskey, Hannah Kesterson, Noereem Z. Mena, Jennifer Dellaport, Laura L. Bellows

**Affiliations:** 1 Department of Food Science and Human Nutrition, Colorado State University, Fort Collins, CO 80532, USA; mlm@colostate.edu (M.L.M.); hannah.kesterson@colostate.edu (H.K.); noereem.mena@colostate.edu (N.Z.M.); 2 Colorado Department of Public Health and Environment, Denver, CO 80246, USA; Jennifer.dellaport@state.co.us

**Keywords:** child, preschool, child day care centers, child health, education, gardens, farms, food

## Abstract

Interest in farm to early care and education (ECE) programming, which consists of gardening, nutrition education, and local food procurement, has been growing in the United States, as it may be a promising technique for promoting healthful foods to young children. However, there is limited information about current farm to ECE efforts in specific states, including Colorado, to support funding and resource needs. An online survey was distributed to licensed Colorado ECE providers in two phases to understand current participation in the farm to ECE as well as provider perspectives on benefits and barriers to programming. A total of 250 surveys were completed. Approximately 60% of ECE facilities participated in gardening and nutrition education with providers almost unanimously agreeing on the child-centric benefits of programming. Fewer facilities (37%) participated in local food procurement likely due to significant time, cost, and knowledge barriers. To increase participation in farm to ECE as a technique for promoting healthful foods to young children, future efforts should focus on innovative solutions to reduce ECE-specific barriers.

## 1. Introduction

Farm to institution initiatives, which consist of partnerships where local producers sell to nearby institutions, have emerged as an opportunity to both promote community health and well-being, and support local economies and agriculture. In the United States (USA), key farm to institution initiatives have consisted of farm to school, farm to hospital, and farm to other large institutional buyers such as universities, government agencies, and corporations [1]. In particular, farm to school programs have been rising in popularity due to the potential benefits of teaching children the origin of the food they eat and promoting fresh, healthy items to youth. In the USA, 42% of school districts participate in farm to school activities by engaging over 23 million students [2]. A recent systematic review examining the effectiveness of farm to school programs and activities reported that studies consistently show positive impacts on student food and nutrition-related knowledge. In addition, most studies suggest a positive relationship between farm-to-school activities and healthy food selection during school meals, nutrition self-efficacy, and willingness to try fruits and vegetables [3]. The impact of farm-to-school activities on fruit and vegetable consumption and preference is unclear [3].

As farm-to-school programs expand, there is growing interest in farm to early care and education (farm to early care and education (ECE)) initiatives, which focus on institutions primarily serving children aged five years and under. In the USA, a majority of children under the age of five attend some form of licensed ECE setting, which makes such settings ideal for focused health promotion efforts in early childhood [4]. Given the evidence indicating the importance of developing healthful food habits early in life [5], farm to ECE programming are viewed as a possible intervention for promoting healthful eating behaviors at a young age [6].

Farm to ECE consists of three key components: gardening, nutrition education, and local food procurement. These components encompass a range of activities, including on-site gardening, educational activities focused on local food such as visits from farmers, trips to a local farm, and serving local foods during meals or snacks [6]. Overall, programming has several goals ranging from providing children with experiential learning opportunities to promoting healthy food items and community engagement. Setting aside the community-level benefits, the stated goal for most farm to ECE programs is to promote health, increase children’s willingness to try new foods, and increase fruit and vegetable consumption [7,8,9,10,11,12,13]. Small, quasi-experimental pilot studies have shown promising trends for gardening’s impact on children’s vegetable consumption [9,12,13] as well as local food procurement and nutrition education on children’s willingness to try new foods [10]. However, more rigorous research on these potential outcomes has been limited to date. A systematic review found for children of all ages that there is strong qualitative evidence showing that gardening has a positive impact on children, but limited quantitative evidence indicating improved vegetable consumption [14]. Similarly, another systematic review on gardening programs for children found that, although there was a small positive influence of gardening on children’s fruit and vegetable intake, many studies were limited by self-reported data and small sample sizes [15].

Despite the need for further research, farm to ECE programming can serve as an example of positive and effective food promotion techniques for healthy foods to young children. Recent systematic reviews and meta-analyses have identified the most effective techniques for developing healthy food preferences, as measured by vegetable intake, in young children: repeated taste exposures, including offering vegetables in their plain form [16], and sensory learning methods, including visual exposure and experiential learning [17]. Additional studies have shown the importance of sensory behaviors [18] incorporating creative and age-appropriate activities into nutrition education [17,19], and that there is a significant positive effect on consumption and willingness to try new foods when nutrition education is combined with engaging gardening and cooking activities [20,21]. If designed appropriately, the components of farm to ECE can synergistically provide fun, age-appropriate nutrition education augmented by gardening activities offering opportunities for sensory exploration and repeated exposure to a variety of fruits and vegetables.

Widespread farm to ECE programming may be an important technique for promoting healthy foods to young children. Therefore, it is important to explore the barriers and facilitators to farm to ECE to understand how best to design interventions, or advocate for policies to support ECE facilities in this work. Although interest in farm to ECE has been growing throughout the U.S., there is limited information related to the prevalence of programming in specific states, including Colorado, to support funding and resource needs. Therefore, the objective of this descriptive study was to understand participation in farm to ECE programming by Colorado ECE licensed providers and to ascertain the perceived benefits and barriers of each of the three farm-to-ECE programming components.

## 2. Materials and Methods

A cross-sectional design was employed to investigate current farm-to-ECE programming in Colorado. An online survey was available to ECE providers for one month at two independent times with response periods separated by approximately one year. Recruitment and participation occurred via email to statewide list surveys. The first survey (Phase 1) was distributed to all Colorado providers participating in the Child and Adult Care Food Program (CACFP) in February 2018. Phase 2 of the survey occurred through two statewide ECE licensed provider e-mail newsletters in January 2019. Due to the online nature of the survey, snowball recruitment occurred as other ECE organizations and state partners shared the survey link with colleagues. Inclusion criteria for the survey were reporting that they worked at an ECE facility in the state of Colorado and were over the age of 18.

The survey consisted of 48 questions, which addressed current practices, barriers, and facilitators to each component of farm-to-ECE (gardening, nutrition education, local food procurement), and captured provider’s facility characteristics. A portion (22) of the survey questions were adapted from the National Farm to School Network Farm to ECE Survey [22,23]. Others were developed by researchers based on an extensive literature review [9,10,12,14,24,25] and Colorado-specific factors (such as a shortened growing season in high altitudes). In order to collect consistent data from all survey participants, a standard definition of farm-to-ECE was provided near the beginning of the survey.

Prior to distribution, the survey was subject to expert review and revision by public health and nutrition professionals to establish content validity. Research Electronic Data Capture (REDCap), a secure web platform for building and managing online databases and surveys, was used data collection and management [26]. Since the survey was built in REDCap, all survey responses were automatically recorded. Responses were required for each question, so if participants completed the survey, there were no missing data. Only completed surveys were used in the analysis. Descriptive statistics were performed as well as a chi-square test of independence to determine if significant relationships existed between farm to ECE activity (e.g., local food procurement (yes or no) and ECE facility characteristics). Data were analyzed using SPSS (SPSS Inc., Chicago, IL, USA) statistics software for Windows, version 25 (IBM Corporation, Armonk, NY). The significance level was set at *p* < 0.05.

## 3. Results

A total of 250 surveys were completed (331 initiated, 75.5% completion rate). The majority of survey respondents were ECE directors (62%), female (96%), and over the age of 40 (72%). The largest proportion of respondents represented public or private child care centers (44%), followed by family homes (29%) and school-based preschool programs (21%). Respondents represented 41 (out of 64) Colorado counties, and were evenly split between an urban (27%), suburban (38%), and rural (36%) location. Nearly half (45%) of survey respondents reported being unfamiliar with the term farm-to-ECE. Centers reported current participation in some form of farm-to-ECE activities (48%) or plans to participate in the future (19%).

### 3.1. Gardening

More than half (59%) of respondents reported that the ECE facility had a garden. Among these facilities, the produce harvested from the garden was used in a variety of ways: 85% of facilities produced meals or snacks for students, 46% sent produce home with families, and 44% incorporated it into nutrition education activities. Five respondents mentioned that the produce harvested from the garden was not of sufficient quantity to be used in any of these activities. The most important benefits of the garden were child-centric with nearly all respondents citing the opportunity to provide experiential learning to children (99.6%), the opportunity for children to try more fruits and vegetables (98.8%), and the opportunity for children to connect with nature (98%) as ‘somewhat’ or ‘very’ important benefits. Challenges associated with gardening were primarily resource limitations, including the cost associated with gardening materials (68%) and limited staff and time to tend to the garden (62%). Most respondents were not concerned about dirt in the classroom (88%) or children’s limited attention spans (70%).

### 3.2. Nutrition Education

A similar number of facilities provided some form of nutrition education (57%). The most popular nutrition education activities related to local food and agriculture were educating children about how food is grown and where it comes from (71%), cooking with children (68%), taste tests of local foods (63%), educating children about locally grown food (40%), and field trips to a local farm or garden (33%). Again, respondents perceived the benefits of nutrition education to be primarily child-centric. Nearly all (99%) indicated helping children develop healthier eating habits and teaching children about how food is grown (98%) were ‘somewhat’ or ‘very’ important benefits. Overall, there were not too many challenges endorsed by respondents with the most challenging factors being that parents are not interested in educational sessions (37%) or the limited time in the day to incorporate the curriculum (35%).

### 3.3. Local Food Procurement

Around one-third of respondents (37%) indicated that their program procured food from local sources. Among those participating in local food procurement, local foods were acquired from multiple places including the grocery store (73%), farmers markets (50%), individual farms (28%), and food hubs (13%). There was a much wider range of perceived benefits and challenges associated with local food procurement when compared to gardening and nutrition education (Table 1 and Table 2). Program type (e.g., school-based preschool program, family home) and enrollment size were associated with local food procurement, (X^2^ (5) = 11.85, *p* = 0.04) and (X^2^ (4) = 16.45, *p* = 0.002), respectively, with smaller and family home facilities being more likely to participate in local food procurement. The summer operation was also associated with local food procurement (X^2^ (1) = 6.84, *p* = 0.009) with facilities that operate during the summer being more likely to participate in local food procurement. Location (urban, suburban, rural), being a school-based preschool program, and participation in a federal program providing reimbursement for meals served in childcare (Child and Adult Care Food Program) were not associated with local food procurement.

### 3.4. Additional Feedback

At the conclusion of the survey, participants were provided with the opportunity to write in additional thoughts related to farm-to-ECE programming. Forty participants wrote short comments that fit into two categories: benefits of programming or challenges and limitations of programming. Among those sharing additional thoughts on benefits, several participants wrote about their belief in the importance of programming, stating that “I honestly believe this is the best thing I have been able to support at my center,” “I have seen how kids will eat the food that they have grown,” and “I love seeing the children gardening and the joy that comes from tasting the food they helped grow.” However, some offered additional insight into barriers to programming as well. Several mentioned cost, stating that “local foods are too expensive in my community,” or that they have to “buy according to grocery store sales.” Others mentioned staff limitations, which involved “many directors and cooks” not having “the knowledge of how to access and prepare fresh food and veggies.” One participant mentioned that statewide health policy ordinances were hindering her ability to take students on trips to farms or have a chicken coop onsite. Collectively, these quotes demonstrate the wide range of barriers to this work.

## 4. Discussion

Overall, rates of participation in farm-to-ECE activities are similar to those reported nationally with 48% of Colorado providers reporting some participation in the last year when compared to 49% nationally [23]. Many Colorado ECE providers are already doing gardening and/or nutrition education, and providers overwhelmingly agree on the child-centric benefits of these two activities. Similar to national surveys [23], providers echoed many of the proposed benefits of farm to ECE programming found in the literature, including the food promotion techniques of experiential learning, multiple opportunities for trying new items, and approaching learning about healthy foods in a fun and creative way. This is promising as it indicates that most providers have the desire and motivation to participate in farm-to-ECE programming and agree that it has significant promise in the promotion of healthy foods to young children.

Although providers endorsed the child-centric benefits, they also cited resource limitations as a key barrier to implementation, which has been seen in other studies [24]. Out of the three components of farm-to-ECE, local food procurement is less frequent in Colorado likely due to the variety of knowledge and logistical barriers, such as knowing how to order local food, how to find suppliers and farmers, delivery considerations, and more. In particular, local food procurement in farm to ECE presents unique challenges compared to other farm-to-institution settings. The quantity of food required for most ECE sites is substantially lower than that of larger institutional settings such as school districts or universities, which suggests ECE sites are often not able to meet the minimum order requirement for a farm or food hub. Additionally, smaller ECE enrollment indicates limited staff, which requires an individual(s) to go beyond normal job duties to champion initiation and/or sustainment of farm-to-ECE efforts, particularly related to sourcing and preparing fresh, local items. These challenges are reflected in the results that show that a majority of ECE providers who procure local foods acquire them from grocery stores or farmers markets and not directly from individual farms or food hubs. These findings are echoed on a national scale with few centers participating in the 2018 national farm-to-ECE survey indicating they procured food from a farmer’s market (34%) or individual farmer/producer (31%) when compared to another source, such as a grocery store or food bank (74%) [23].

Therefore, increasing participation in farm-to-ECE programming may require innovative models and solutions. For example, partnerships with local agricultural experts might be helpful in setting up gardens or working with community-level volunteer groups (i.e., Cooperative Extension Master Gardeners) to tend to the garden could help lower the burden on ECE staff. To increase local food procurement, creative solutions include partnerships between ECE providers to create bulk orders to meet minimum purchasing requirements of farmers or food hubs, or facilitating community-level networking and training events to bring together farmers and ECE staff. Current efforts are underway in Southern Colorado to test the feasibility of these types of models, including a pilot project linking ECE centers to submit weekly orders to a local food hub that meets the minimum weekly purchase requirement and networking efforts to build partnerships between interested farmers and ECE facilities.

This study is not without limitations, including a relatively low sample size and possible selection bias in respondents, if those who had an existing interest in farm-to-ECE were more likely to participate. This may have resulted in a sample with greater farm-to-ECE participation, even though participation rates were similar to those seen nationally [23]. Since the goal was to capture information specific to Colorado, the results may not be generalizable to other locations. However, many of the benefits and barriers identified by providers are broadly applicable and reflect trends seen in the national farm-to-ECE survey [23]. Although a majority of survey respondents were female, this reflects the landscape of the childcare workforce in the United States and providers who are enthusiastic about farm to ECE may be able to expand this work to their personal lives and broader communities. A key strength of this study was that it provided additional insight and detail into participation and provider perspectives on farm to ECE in Colorado, which have been used to inform multiple pilot projects. Respondents represented a range of facilities and locations across Colorado.

## 5. Conclusions

Overall, providers are enthusiastic about farm-to-ECE programing, but need additional supports and creative solutions to fully implement all components. Farm-to-ECE programming offers promotion techniques of healthy foods via experiential learning activities coupled with multiple opportunities for repeated taste exposure and sensory exploration, which can build children’s familiarity with new foods and increase their likelihood of trying new foods, which are important factors for food acceptance and healthful diets [17,27,28]. However, further research, including well-designed experimental studies assessing the impact of farm-to-ECE programs on children’s vegetable consumption, is critical to solidify the evidence for farm-to-ECE programming as a tool for promoting healthy foods to young children. Although there are clear techniques by which farm to ECE could lead to an improvement in healthy behaviors and increased consumption of healthy foods, more objective and rigorous research is necessary to confirm that these approaches are effective for increasing young children’s consumption of fruits and vegetables.

## Figures and Tables

**Table 1:** Perceived benefits of local food procurement among licensed Colorado Early Care and Education (ECE) providers ^1^.

How Important are Each of the Following Possible Benefits Related to Procuring Local Food?	Very Important (%)	Somewhat Important (%)	Neither (%)	A little Important (%)	Not at All Important (%)
Supporting local farmers	71.2	20.4	7.6	0.8	0
Access to higher quality food	69.6	22.8	6.0	1.2	0.4
Supporting local economy and community	68.8	24.8	6.0	0.4	0
Access to fresher food	66.4	26.0	4.4	2.8	0.4
Local food is more nutritious	57.2	24.8	14.8	1.6	1.6
Local food tastes better	56.8	26.0	12.8	2.8	1.6
Appeals to parents	38.8	35.2	20.0	3.6	2.4

^1^*N* = 250 unless otherwise specified.

**Table 2:** Perceived challenges of local food procurement among licensed Colorado Early Care and Education providers ^1^.

How Challenging are Each of the Following Factors Related to Procuring Local Food?	Very Challenging (%)	Somewhat Challenging (%)	Neither (%)	A Little Challenging (%)	Not at All Challenging (%)
Cost/price of items	44.0	35.6	8.0	6.8	5.6
Finding suppliers/farmers	22.0	33.2	14.4	18.0	12.4
Inadequate storage space	21.6	35.6	19.2	8.0	15.6
Delivery considerations	21.2	42.4	13.2	13.2	10.0
Seasonality of fruits and vegetables	21.2	45.2	13.2	11.6	8.8
Sink capacity too small or can’t handle soil ^2^	21.2	19.4	20.0	13.9	25.5
Time to prepare fresh foods ^2^	20.0	30.3	10.9	18.8	20.0
Knowing how to order local products	19.2	40.0	15.6	14.4	10.8
Access to kitchen equipment to prepare foods	14.8	14.8	21.2	9.2	40.0
Food safety	14.4	24.4	25.2	15.6	20.4
Quantity and type of foods ^2^	13.9	32.7	19.4	15.8	18.2
Unreliable supply	13.2	41.6	20.4	17.6	7.2
Quality	9.6	27.2	26.0	16.0	21.2

^1^*n* = 250 unless otherwise specified. ^2^ Only included in Phase 2 of survey, *n* = 165.

## References

[1] United States Department of Agriculture Know Your Farmer Know Your Food Compass: Farm to Institution Initiatives. https://www.usda.gov/sites/default/files/documents/6-Farmtoinstitution.pdf.

[2] USDA Farm to School Census. https://farmtoschoolcensus.fns.usda.gov/.

[3] Prescott M.P., Cleary R., Bonanno A., Costanigro M., Jablonski B.B.R., Long A.B. (2019). Farm to School Activities and Student Outcomes: A Systematic Review. Adv. Nutr..

[4] Benjamin-Neelon S.E. (2018). Position of the Academy of Nutrition and Dietetics: Benchmarks for Nutrition in Child Care. J. Acad. Nutr. Diet..

[5] Luque V., Escribano J., Closa-Monasterolo R., Zaragoza-Jordana M., Ferré N., Grote V., Koletzko B., Totzauer M., Verduci E., ReDionigi A. (2018). Unhealthy Dietary Patterns Established in Infancy Track to Mid-Childhood: The EU Childhood Obesity Project. Nutrition.

[6] Hoffman J.A., Schmidt E.M., Wirth C., Johnson S., Sobell A.S., Pelissier K., Harris D.M., Izumi B.T. (2017). Farm to Preschool: The State of the Research Literature and a Snapshot of National Practice. J. Hunger Environ. Nutr..

[7] Lee R.E., Lorenzo E., Szeszulski J., Arriola A., Bruening M., Estabrooks P.A., Hill J., Marsiglia F.F., O’Connor T., Pollins K.S. (2019). Design and methodology of a cluster-randomized trial in early care and education centers to meet physical activity guidelines: Sustainability via Active Garden Education (SAGE). Contemp. Clin. Trials.

[8] Sharma S.V., Hedberg A.M., Skala K.A., Chuang R.-J., Lewis T. (2014). Feasibility and acceptability of a gardening-based nutrition education program in preschoolers from low-income, minority populations. J. Early Child. Res..

[9] Brouwer R.J.N., Neelon S.E.B. (2013). Watch Me Grow: A garden-based pilot intervention to increase vegetable and fruit intake in preschoolers. BMC Public Health.

[10] Izumi B.T., Eckhardt C.L., Hallman J.A., Herro K., Barberis D.A. (2015). Harvest for Healthy Kids Pilot Study: Associations between Exposure to a Farm-to-Preschool Intervention and Willingness to Try and Liking of Target Fruits and Vegetables among Low-Income Children in Head Start. J. Acad Nutr. Diet.

[11] Dannefer R., Power L., Berger R., Sacks R., Roberts C., Bikoff R., Solomon E. (2018). Process evaluation of a farm-to-preschool program in New York City. J. Hunger Environ. Nutr..

[12] Farfan-Ramirez L., Diemoz L., Gong E.J., Lagura M.A. (2011). Curriculum Intervention in Preschool Children: Nutrition Matters!. J. Nutr. Educ. Behav..

[13] Kalich K., Bauer D., McPartlin D. (2009). Early Sprouts: Cultivating Healthy Food Choices in Young Children.

[14] Ohly H., Gentry S., Wigglesworth R., Bethel A., Lovell R., Garside R. (2016). A systematic review of the health and well-being impacts of school gardening: Synthesis of quantitative and qualitative evidence. BMC Public Health.

[15] Savoie-Roskos M.R., Wengreen H., Durward C. (2017). Increasing Fruit and Vegetable Intake among Children and Youth through Gardening-Based Interventions: A Systematic Review. J. Acad. Nutr. Diet..

[16] Nekitsing C., Blundell-Birtill P., Cockroft J.E., Hetherington M.M. (2018). Systematic review and meta-analysis of strategies to increase vegetable consumption in preschool children aged 2–5 years. Appetite.

[17] Nekitsing C., Hetherington M.M., Blundell-Birtill P. (2018). Developing Healthy Food Preferences in Preschool Children Through Taste Exposure, Sensory Learning, and Nutrition Education. Curr. Obes. Rep..

[18] Moding K.J., Bellows L.L., Grimm K.J., Johnson S.L. (2020). A longitudinal examination of the role of sensory exploratory behaviors in young children’s acceptance of new foods. Physiol. Behav..

[19] Bellows L., Anderson J. (2006). The food friends: Encouraging preschoolers to try new foods. Young Child..

[20] Jaenke R.L., Collins C.E., Morgan P.J., Lubans D.R., Saunders K.L., Warren J.M. (2011). The Impact of a School Garden and Cooking Program on Boys’ and Girls’ Fruit and Vegetable Preferences, Taste Rating, and Intake. Health Educ. Behav..

[21] McAleese J.D., Rankin L.L. (2007). Garden-Based Nutrition Education Affects Fruit and Vegetable Consumption in Sixth-Grade Adolescents. J. Am. Diet. Assoc..

[22] Stephens L., Oberholtzer L. (2016). Results from the 2015 National Survey of Early Care and Education Providers: Local Procurement, Gardening, and Food and Farm Education.

[23] Shedd M., Stephens L., Matts C., Laney J. (2018). Results from the 2018 National Farm to Early Care and Education Survey.

[24] Davis K.L., Brann L.S. (2017). Examining the Benefits and Barriers of Instructional Gardening Programs to Increase Fruit and Vegetable Intake among Preschool-Age Children. J. Environ. Public Health.

[25] Kos M., Jerman J. (2012). Preschool children learning about the origin of food, on local farms and in the preschool garden. Nutr. Food Sci..

[26] Harris P.A., Thielke R., Payne J., Gonzalez N., Conde J.G. (2009). Research electronic data capture (REDCap)—A metadata-driven methodology and workflow process for providing translational research informatics support. J. Biomed. Inform..

[27] Holley C.E., Farrow C., Haycraft E. (2017). A Systematic Review of Methods for Increasing Vegetable Consumption in Early Childhood. Curr. Nutr. Rep..

[28] Johnson S.L., Ryan S.M., Kroehl M., Moding K.J., Boles R.E., Bellows L.L. (2019). A longitudinal intervention to improve young children’s liking and consumption of new foods: Findings from the Colorado LEAP study. Int. J. Behav. Nutr. Phys. Act..

